# Advances in Hepatic Tissue Bioengineering with Decellularized Liver Bioscaffold

**DOI:** 10.1155/2019/2693189

**Published:** 2019-05-06

**Authors:** Erik Aranha Rossi, Luiz Fernando Quintanilha, Carolina Kymie Vasques Nonaka, Bruno Solano de Freitas Souza

**Affiliations:** ^1^Escola de Ciências da Saúde-Universidade Salvador-UNIFACS, Salvador, BA, Brazil; ^2^São Rafael Hospital, D'Or Institute for Research and Education (IDOR), Salvador, BA, Brazil; ^3^Gonçalo Moniz Institute, Fiocruz, Salvador, BA, Brazil; ^4^Faculdade de Tecnologia e Ciências-FTC, Salvador, BA, Brazil; ^5^National Institute of Science and Technology for Regenerative Medicine, Rio de Janeiro, RJ, Brazil

## Abstract

The burden of liver diseases continues to grow worldwide, and liver transplantation is the only option for patients with end-stage liver disease. This procedure is limited by critical issues, including the low availability of donor organs; thus, novel therapeutic strategies are greatly needed. Recently, bioengineering approaches using decellularized liver scaffolds have been proposed as a novel strategy to overcome these challenges. The aim of this systematic literature review was to identify the major advances in the field of bioengineering using decellularized liver scaffolds and to identify obstacles and challenges for clinical application. The main findings of the articles and each contribution for technique optimization were highlighted, including the protocols of perfusion and decellularization, duration, demonstration of quality control—scaffold acellularity, matrix composition, and preservation of growth factors—and tissue functionality after recellularization. In previous years, many advances have been made as this technique has evolved from studies in animal models to human livers. As the field develops and this promising technique has become much more feasible, many challenges remain, including the selection of appropriate cell types for recellularization, route of cell administration, cell-seeding protocol, and scalability that must be standardized prior to clinical application.

## 1. Introduction

Liver diseases, including cirrhosis and hepatocellular carcinoma, remain among the main causes of global mortality [1]. Despite intense international effort, liver transplantation continues to be the only available therapeutic option for end-stage liver disease, which is a procedure with several inherent limitations [2]. Recent data indicate that the demand for liver transplants, in some countries, is nearly three times the number of transplants performed. Furthermore, significant numbers of patients still die while in the transplant waiting list, demonstrating the urgent need for the development of alternative therapies [3, 4].

Recently, whole-organ bioengineering has been proposed as a promising alternative to overcome the challenges involved in liver transplantation, including organ shortage and immune rejection. One approach is to produce a natural bioscaffold through liver decellularization. This technique consists of removing liver cells by perfusion with enzymes and/or detergent solutions, or by physical methods, to generate extracellular matrix- (ECM-) derived scaffolds while preserving vascular integrity. This is followed by the introduction of new cells with the appropriate characteristics and repopulation potential [5]. The efficiency and functionality of the bioengineered liver tissue can be tested by evaluating specific biomarkers. A key advantage of using a decellularized liver bioscaffold is the preservation of liver-specific ECM, architecture, and bioactive molecules, thus providing the necessary signals for hepatocyte engraftment, survival, and function [6].

Recent studies have shown promising results in the field of liver tissue bioengineering, augmenting the speculation that a fully functional liver tissue could be generated ex vivo, potentially offering an alternative for liver transplantation [7]. Tissue engineering using a decellularized liver scaffold is a relatively new technique, which remains under development despite significant protocol optimizations and improvements in recent years. The aim of this integrative literature review was to identify the major advances in the field of bioengineering using decellularized liver scaffolds and identify bottlenecks for clinical translation.

## 2. Methods

### 2.1. Search Strategies

This is an integrative review of the literature, adapted from the PRISMA (Preferred Reporting Items for Systematic Reviews and Meta-Analyses) guide. The databases SciELO (http://www.scielo.org), PubMed (http://www.ncbi.nlm.nih.gov/pubmed), and LILACS (http://lilacs.bvsalud.org) were searched between December 2017 and March 2018. An additional search using references contained in the main selected articles was also conducted. Searches were performed using the descriptive terms “liver,” “recellularization,” and “decellularization” based on the medical terms of the National Library of Medicine (https://meshb.nlm.nih.gov/search). The Boolean expression “AND” was used in order to find the registries in which “liver” was associated with at least one other descriptive term listed.

### 2.2. Study Selection

Initially, literature searches were performed independently by the authors of the current study, which were followed by analysis of the identified article titles and abstracts in order to confirm that the publication contained detailed descriptions of protocols for hepatic tissue decellularization (with or without recellularization and in vivo transplant). Selected articles were then read in their entirety in order to compose the current study. The inclusion criteria were as follows: experimental studies, published in the past ten years, aiming to produce a natural bioscaffold, and through liver decellularization. The included articles describe methods capable of sustaining adequate liver repopulation, viability, and function of the bioengineered tissue. No language restriction was applied. The following exclusion criteria were considered: review articles, letters, and conference abstracts.

Relevant information, such as journal impact factors, publishing year, proposed methods, results, and innovations, was also considered. Some of the investigated studies are described with greater details in Results, while others are used in table formats with described information regarding their main contributions.

## 3. Results

### 3.1. Literature Searches and Inclusion Assessment

**Figure 1:**
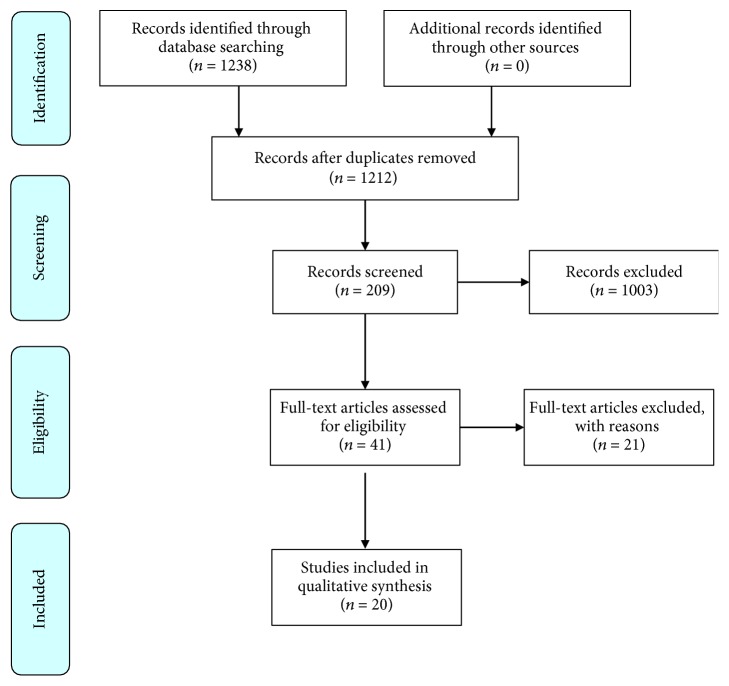
Fluxogram of the literature review demonstrating articles that were identified and selected at each step.

**Figure 2:**
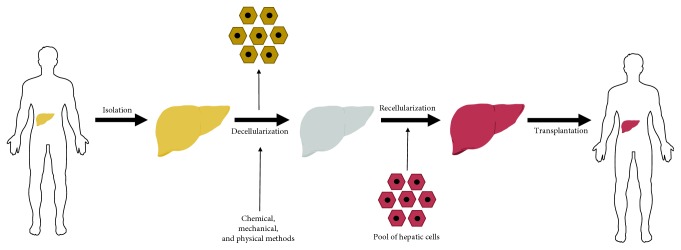
General concept and steps for the generation of bioengineered liver tissue. Organs that are nonviable for transplantation may serve as the basis for the generation of a scaffold that can then be repopulated with liver cells for subsequent transplantation in patients with liver disease.

**Figure 3:**
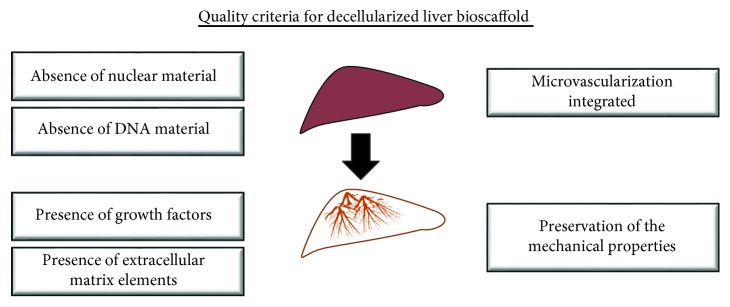
Quality control criteria for the evaluation of successful decellularization.

Compiling the results from searches performed in all databases, 1238 articles were found. By analyzing titles and abstracts, we identified articles that were repeated in more than one database, while others did not fit with the criteria established for this study. Finally, 20 articles were included in the review (Figure 1). The articles were published in English between 2010 and 2018, in international journals. All articles used either one method or a combination of different strategies: perfusion with chemicals, enzymatic reagents, or physical methods. A general scheme for the procedures performed in the production of bioengineered liver tissue is presented in Figure 2. Quality criteria generally acceptable and utilized in the studies are also summarized (Figure 3).

### 3.2. General Overview of Methods

The experiments were conducted mainly in the rat liver [8–19], as well as in mouse [20], pig [21–24], sheep [19], ferret [11], minipig [25], and human [26, 27] liver tissues. Perfusion was performed through the hepatic vasculature system, which consists of four major vessels, the portal vein (PV), hepatic artery (HA), inferior vena cava (IVC), and superior vena cava (SVC). While PV is the most common route for perfusion decellularization [8, 10–12, 16, 18, 20, 22, 25], perfusion via IVC [9, 13, 24, 26, 27], SVC [15], HA [21], or utilizing PV and HA simultaneously [19, 23] has been also performed. For characterization of the bioscaffold, the studies frequently used H&E staining [8–23, 25–27], evaluation of matrix composition by immunostaining or by other methods [8–14, 17–27], and ultrastructural analysis by electron microscopy [10–14, 16–22, 25–27]. Confirmation of acellularity and elimination of nuclear DNA were also evaluated, which are crucial to reduce graft immunogenicity. Two studies applied nondestructive imaging methods for the evaluation of the scaffold's structure: ultrasound [16] and 3D-computed tomography scanning [23]. Eight studies evaluated and quantified the preservation of growth factors in a decellularized bioscaffold [12, 13, 15, 17, 18, 22, 24, 27]. Four studies did not perform the recellularization step [5, 12, 23, 24]. Regarding the cell types used in the recellularization step, seven studies used primary hepatocytes [8, 10, 13–15, 18, 27], three studies used liver progenitor cells [9, 11, 19], and six studies used immortalized hepatocytes and/or nonparenchymal cell lines [16, 17, 21, 22, 26, 27]. Two studies investigated the potential use of mesenchymal stem cells (MSCs)—either undifferentiated or stimulated to undergo hepatic differentiation—as an extrahepatic source for recellularization alone or in association with hepatocytes [15, 20]. Four studies performed an additional *in vivo* validation step by transplanting the bioengineered liver tissue into experimental animals [8, 14, 21, 26]. The main findings of such studies are described chronologically and summarized in Table 1.

### 3.3. Evolution of Decellularization Methods

The goal of the liver decellularization step is to provide an acellular scaffold while maintaining the original chemical and biological components of the tissue, thus providing an adaptable environment for cultured cell maturation and functionality. However, the use of physical, chemical, and biological methods, in combination or separately, can potentially cause disruption of original tissue characteristics. In the latest years, several technical advances have occurred, allowing for the generation of a well-preserved decellularized liver bioscaffold.

The first report of the generation of a decellularized liver bioscaffold was published in 2010, by adaptation of a previous study that performed heart tissue decellularization [28]. Uygun and colleagues used sodium dodecyl sulfate (SDS), an anionic detergent that causes cell lysis and solubilizes cytoplasmic components, for decellularization of liver tissue. The procedure was performed in Lewis rats, over a duration of 72 hours. Following protocol conclusion, immunostaining assays revealed the maintenance of the native matrix characteristics, composed primarily of collagens type I and IV, fibronectin, and laminin-*β*1. Additionally, matrix acellularity was confirmed by DAPI staining. Staining with Allura Red demonstrated that the microvasculature was also preserved [8]. The extended duration of the procedure, however, could limit scalability and the preservation of growth factors bound to the ECM, which was not evaluated.

In another study from 2010, Shupe and colleagues presented a simpler and efficient decellularization technique and achieved consistent results in a less time-consuming process by combining perfusion with Triton X-100 and 0.1% SDS, resulting in the DNA removal, assessed by DAPI staining. Immunostaining was performed in order to demonstrate the presence of type IV collagen in the matrix. Laminin was also present in the venous reminiscent basal membrane and in the surrounding acellular remnants of the hepatic cords [29].

At this point, the described protocols had long durations, which may compromise the scaffold quality, due to the loss of key liver ECM components, such as matrix-bound growth factors. De Kock and colleagues presented a simple and rapid method for decellularization of whole rat livers, achieving a drastic reduction in the procedure duration, which lasted for only 60 minutes. De Kock's study was performed in Sprague-Dawley rats, by perfusing the liver for 30 minutes with 1% Triton X-100 solution followed by 30 minutes with 1% SDS solution at 37°C and 30 ml/min flow rate, a 30-fold higher flow rate, compared to the previous protocols. A translucent, acellular scaffold was obtained, with acellularity confirmed by scanning electron microscopy, which demonstrated the absence of cells in the newly decellularized liver matrix. Similar to the above-mentioned protocols, maintenance of most ECM proteins (type I and IV collagens, fibronectin, and laminin) was observed, indicating the preservation of the structure and components of the basement membrane. In addition, the authors demonstrated the presence of vascular endothelial growth factor (VEGF) directly attached to the ECM, in association with large blood vessels and sinusoidal spaces. The absence of positive staining for hematoxylin indicated that the decellularization process was successful and efficiently achieved a drastic reduction in the procedure time [12].

Based on a previously described protocol [30], Gessner and colleagues used the VC for removing fluids and the portal vein to perfuse the detergents. A delipidation buffer (36 U/l of phospholipase A2 in 1% sodium deoxycholate) was infused until the tissue became transparent. Additionally, in order to maintain the biological and chemical characteristics of the tissue, the authors perfused the liver with a high-salt buffer, which favors the maintenance of collagen in an insoluble state, while also preserving cytokines and growth factors bound to them. DNase and RNase were used to remove any nucleic acid that remained in the framework. Scanning electron microscopy (SEM) images showed tissue preservation at comparable levels to normal liver tissue. Notably, the protocol also was associated with increased maintenance of the microvasculature integrity as evaluated by ultrasound, without the need to use dyes, which is relevant for future translational studies [16].

Also, in 2013, Yagi and colleagues presented an important improvement of the decellularization technique. The authors adapted the protocols proposed by Uygun et al. [8] and Shupe et al. [9] and applied them in larger animal studies, a crucial step for further clinical translation. The authors performed the procedure in pig livers, which are of similar size as those of human livers. After the procedure, DNA was not detected and the morphological and structural components were preserved. Growth factors such as hepatocyte growth factor (HGF), basic fibroblast growth factor (bFGF), VEGF, and insulin-like growth factor 1 (IGF-1), essential for conditioning a healthy niche to hepatic cells, were evaluated and detected, however in significantly lower levels, compared to normal liver tissue [22].

Continuing in the development of potential clinical applications, Struecker et al. [23] presented a technique to decellularize pig livers in seven hours, a much lower duration than that which was previously reported in large animal livers. Using the pressure control method and perfusing with 1% of Triton X-100 and 1% of SDS through the HA (120 mmHg) and PV (60 mmHg), the efficiency of the technique was observed by macroscopic observation, histological staining (H&E, Sirius Red, and Alcian blue), immunohistochemical staining (for collagen IV, laminin, and fibronectin), biochemical evaluation (DNA, collagen, and glycosaminoglycans), and verification of microvasculature integrity by three-dimensional computed tomography. The authors proposed that the organs decellularized with pressure oscillation were more homogeneous during the process and presented less residual DNA, with no remaining cells and no changes in ECM [23].

With efforts to get closer to clinical applicability, Mazza and colleagues performed for the first time, in 2015, the decellularization of a human liver. The liver tissues were frozen at -80°C and thawed at 4°C. The perfusion protocol used by Mazza and colleagues consisted of subjecting the hepatic tissue to infusions with different solutions, including distilled water, trypsin/EDTA, SDS, Triton X-100, saline, peracetic acid, and ethanol in a protocol that lasted for up to 6 weeks. Histological analysis was performed to demonstrate acellularity and DNA quantification, while immunohistochemical analysis showed that the main components of the ECM were preserved [26].

Again, in 2017, Maaza et al. improved the decellularization technique based on an oscillation of the g-force and high shear stress [27]. The liver was frozen at -80°C, thawed at 4°C, cut into 125 mm^3^ cubes, and frozen again at -80°C. The cubes were then thawed in a water bath (37°C) for 1 hour and covered with 1% PBS. After being thawed, they were transferred to 2 ml tubes, detergents were added, and different g-force values were tested. The macroscopic analysis of the tissues showed a translucent and transparent appearance, while the H&E staining confirmed the removal of nuclear material with preservation of the ECM, as demonstrated by the stains of Sirius Red and Elastin Van Gieson. The DNA quantification was also measured to be below 50 ng/mg, which is the preferred method for evaluation of contamination with DNA. Moreover, the protocol time was dramatically reduced from 36 hours to approximately 3 hours [27].

### 3.4. Recellularization Efforts

The final outcome of a decellularized liver bioscaffold is to serve as the basis for the recellularization, resulting in a viable and functional tissue for in vitro tests and transplantations. The first reported attempt to recellularize a liver scaffold was made by Uygun et al. in 2010 [8]. To test the effectiveness of the liver scaffold generated, a recellularization protocol was tested using four infusions of 5 × 10^6^ rat primary hepatocytes through the PV. The engraftment efficiency was estimated as 95.6%±3.4%. Initially, the transplanted cells were localized around large veins, and in the subsequent days, the cells were observed to be distributed throughout the entire matrix; however, approximately 20% were found to be undergoing cell death by apoptosis. During the evaluated time frame, biochemical analysis demonstrated increased expression levels of UDP glucuronosyltransferase 1 family, polypeptide A1, glucose 6-phosphatase, albumin, and urea. The expression levels of cytochrome P450 enzymes were reported to be similar to those found in normal livers. The study also tested the addition of microvascular endothelial cells in the repopulated hepatocyte tissue, which were able to align the vasculature in three days.

Shupe and colleagues tested recellularization with a rat liver progenitor cell, WB344. After a total of 10^6^ cells were perfused in tissue through the IVC, the authors were able to see that these cells could migrate from the vessels, which had their structure maintained, to the center of the matrix [9]. Long-term analyses were not performed. Later, Gessner et al. reseeded the liver bioscaffold with human hepatoblast-like cells, Hep3B cells (1.3 × 10^8^ cells). To measure the efficacy of this protocol, SEM was performed and showed engraftment of these cells in the matrix scaffold. Additionally, the engrafted cells presented proliferation potential (Ki67 staining) with no evidence of apoptosis. Albumin and EpCAM were both expressed but in different levels depending of the localization of the reseeded cells [16].

Yagi and colleagues also were able to achieve some success in the cell engraftment of a porcine liver scaffold. A total of 1 × 10^9^ hepatocytes were introduced through PV in a basal medium supplemented with epidermal growth factor (EGF), hydrocortisone, insulin, glucagon, and antibiotics. In the first 24 hours, the hepatocytes were retained in the portal vein but gradually migrated and engrafted in the liver parenchymal in the following days. On the fourth day, grafted hepatocytes presented similar levels of albumin expression, when compared to normal livers. The levels of protein synthesis, as measured by the presence of albumin and the concentration of urea, were slightly higher than the culture of hepatocytes grown in collagen-coated plates. However, long-term functionality of the recellularized liver tissue was not determined and may not occur, since albumin expression dropped considerably after the seventh day of culture [22].

In contrast to the use of parenchymal and nonparenchymal liver cells, Jiang and colleagues used the liver scaffold to provide an environment to support hepatic differentiation of mesenchymal stem cells (MSC). This strategy proved to be better than 2D culture, based on the expression of hepatic-associated genes, marker proteins, glycogen storage, albumin secretion, and urea production. Using the liver scaffold to aid hepatic differentiation of alternative cell sources (for example, stem cells) could be a beneficial tool for clinical application as this study showed in an experimental model of acute liver failure induced by carbon tetrachloride [20].

In another study by the same authors using human decellularized liver, Mazza and colleagues applied four cell types in a sterilized decellularized tissue. Human umbilical vein endothelial cells (HUVECs), human hepatic stellate cell lines (LX2), human hepatoblastoma cell lines (HepG2), primary hepatocytes, and stellate cells were used, and several factors related to the hepatic environment were investigated over the next 3 to 14 days. Immunostaining confirmed migration, attachment, and functionality of HUVECs in the liver scaffold. Expression of important growth factors, such as platelet-derived growth factor beta receptor (PDGF*β*-R) and transforming growth factor beta receptor (TGF*β*-F) was also evaluated. Functionally, quantitative RT-PCR revealed higher albumin expression levels when compared to the 2D culture system. These results allowed the authors to conclude that the heterogeneous system in a developed microenvironment in vitro can mimic a physiologically and anatomically healthy liver and provide the necessary stimuli for the production of a laboratory-developed organ [26, 27].

Despite the improvements seen in recellularization, in vitro functional liver tissue has yet to be efficiently achieved. Additionally, there is no consensus about which protocol provides the best infusion route, cell type, cell quantity, and culture procedures. Significant improvements would be necessary, also including nonparenchymal cells, such as liver sinusoidal endothelial cells, stellate cells, biliary epithelial cells, and Kupffer cells, in order to improve tissue functionality.

## 4. Discussion

**Figure 4:**
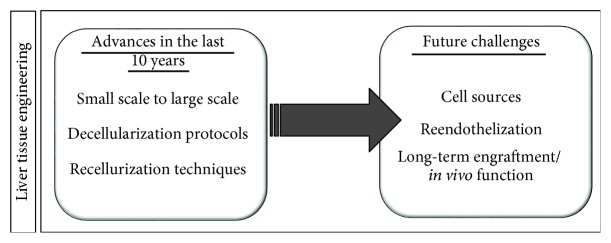
Advances and future challenges for the development of clinically relevant bioengineered liver tissue.

In recent years, many advances in liver decellularization protocols have been made, including scaling up of the application procedures from small to larger animals. However, challenges still remain, both involving the decellularization and, mainly, recellularization steps (Figure 4). Moreover, successful transplantation and viability of the bioengineered scaffold will depend largely on reendothelization and integration to the host vasculature.

Regarding the choice of the detergent used in the decellularization step, these reviewed studies demonstrated that SDS is more effective for DNA removal but can also alter significantly the matrix composition [25, 31, 32]. Therefore, the use of Triton X-100 has been associated with the improved function of the bioengineered liver tissue. While different routes have been tested for the perfusion with the selected solutions in the decellularization step, there is evidence to suggest that HA cannulation, associated with oscillating pressure, may be more efficient [33]. However, there continue to exist protocol efficiency limitations and thus a need to standardize the minimal acceptable level of quality control when evaluating the decellularized liver bioscaffold.

An important issue not always commented on by the studies is the protocols for decontamination and sterilization of the bioscaffold. It should be noted that the ideal method choice should take into consideration the possibility of such methods to alter matrix characteristics, which were previously shown for high gamma irradiation in a porcine dermal bioscaffold. There is evidence to suggest that the combination of peracetic acid with gamma irradiation and ethylene oxide gas may sustain the mechanical properties of the scaffold [34].

Regarding the recellularization step, the ideal cell types to be used for production of a bioengineered liver are not established. Studies have focused in using hepatocytes, which, in order to be obtained for clinical applications, would require human livers, which are scarce. Therefore, extrahepatic sources for liver repopulation would be highly desired. So far, a few studies have used MSCs, but those cells have limited plasticity and their ability to generate fully functional hepatocytes is questionable [15, 20]. These cells could be associated with other cell types to increase graft survival due to their immunomodulatory and trophic paracrine actions [35]. Other potential sources would be hepatocyte-like cells derived from human embryonic or induced pluripotent stem cells (iPSCs), which have been successfully applied and seeded into a decellularized porcine liver bioscaffold [36]. Also, the association with cholangiocytes, endothelial cells, and other nonparenchymal liver cells would be required for adequate function of the engineered liver. All of those cell types could be potentially generated from pluripotent stem cells [37, 38].

Beyond the cell-type choice, the recellularization protocol has yet to be standardized to uniformly repopulate the scaffold, and therefore, there is still much need for optimizations. Reendothelization is a critical step that should be further improved in order to prevent coagulation and graft loss after in vivo transplantation, since the exposure of liver ECM to blood triggers coagulation. Based on this, it may be necessary to use more than one access point for perfusion with endothelial cells, as previously demonstrated [7].

Importantly, reendothelization of the whole-porcine liver was achieved, with vascular patency and absence of coagulation in vivo [39]. The association with extracellular matrix components can also increase the efficiency of reendothelization. Recently, immortalized endothelial cells were utilized in a proof-of-concept study that demonstrated that the association with gelatin enhances reendothelization of a decellularized liver scaffold [40]. The attachment of endothelial cells was also improved by conjugation with a biopolymer, REDV-ELP, which was able to increase reendothelization of a rat decellularized liver bioscaffold [41]. Although these results are promising, both studies use immortalized endothelial cells, and since primary liver endothelial cells cannot be obtained and cultured easily, iPSC-derived endothelial cells could be useful as alternative cell sources in further studies.

Finally, the present study provided an integrative literature review which has limitations, including the diversity of study designs and the variability of the reported protocols and outcome measurements. Moreover, all of the studies reported positive results, which could possibly be explained by publication bias, leading negative or neutral results to not be published.

## 5. Conclusion

As the result of international efforts, the literature demonstrates that clear progress has been achieved in the liver decellularization technique. The recellularization/reendothelization steps, however, still require considerable further development. Nevertheless, the advances to date have made it possible to develop a bioartificial liver much more realistically, aiming at future clinical applications in the hepatology field.

## Figures and Tables

**Table 1:** Summary of protocols and main findings of studies in the liver bioengineering field.

References	Experimental model	Decellularization methods	Recellularization methods	Major contributions
Uygun et al., USA [8]	Female Lewis rats (150–200 g)	Protocol (i) Physical (ii) Chemical Quality assessments (i) Maintenance of ECM components and architecture: collagen and GAG content; immunostaining (ii) Acellularity/absence of nuclear content: histological evaluation and DAPI staining (iii) Maintenance of vasculature: Allura Red AC dye and scanning electron microscopy (SEM)	(i) Infusion of 50 × 10^6^ of rat primary hepatocytes, through PV (ii) 40 × 10^6^ microvascular endothelial cells through PV (iii) Biochemical analysis: albumin, urea, G6P, Ugt1a, and others	The first description of a protocol to generate a liver bioscaffold. Efficient recellularization was achieved supporting liver-specific function (albumin secretion, urea synthesis, and cytochrome P450) for a further transplantation

Shupe et al., USA [9]	Fisher 344 rats (not reported)	Protocol (i) Chemical Quality assessments (i) Maintenance of ECM components and architecture: immunohistochemical staining (ii) Acellularity/absence of nuclear content: DAPI staining (iii) Maintenance of vasculature: immunohistochemistry of laminin	(i) Infusion with WB344 rat liver progenitor cells through the IVC	A relatively simple method to decellularize a whole rat liver without removing the organ is presented

De Kock et al., Belgium [12]	Sprague-Dawley rats (250 g–300 g)	Protocol (i) Chemical Quality assessments (i) Maintenance of ECM components and architecture: immunostaining (ii) Acellularity/absence of nuclear content: SEM/hematoxylin and DAPI staining (iii) Biochemical parameters: VEGF immunostaining	Not performed	An effective and faster method of liver decellularization is presented

Soto-Gutierrez et al., USA [13]	Sprague-Dawley rats (250 g–300 g)	Protocol (i) Physical (ii) Chemical (iii) Enzymatic Quality assessments (i) Maintenance of ECM components: immunostaining and SEM (ii) Acellularity/absence of nuclear content: DNA quantification, H&E, and DAPI staining (iii) Biochemical parameters: bFGF/VEGF/HGF immunoassays (iv) Maintenance of vasculature: transmission electron microscopy (TEM), blue dye	(i) 10 to 50 × 10^6^ primary mouse hepatocytes (ii) Three ways to seed cells: (a) direct parenchymal injection, (b) continuous perfusion, or (c) multistep infusion (iii) SEM showed hepatocyte engraftment (iv) Ki67 showed proliferating cells in recellularized liver (v) Functionality was measured by albumin production, ammonia metabolism, and CYP1A1/2 activity	Decellularization was accomplished in 48 h without the use of harsh detergents preserving 30-50% of growth factor content. It presents a perfusion technique (multistep infusion) resulting in ~90% grafting efficiency with maintenance of some liver functionalities

Bao et al., China [14]	Lewis rats (180–250 g)	Protocol (i) Chemical Quality assessments (i) Maintenance of ECM components and architecture: histological evaluation (ii) Acellularity/absence of nuclear content: SEM and DAPI staining	(i) 1 × 10^8^ rat hepatocytes with spheroid formation (ii) Pretreatment with heparin (iii) Further transplantation	It is reported that the use of hepatocyte spheroids increases cell survival. Tissue-engineered liver can maintain hepatic functions up to 72 hours

Gessner et al., USA [16]	Wistar rats (250–300 g)	Protocol (i) Chemical (ii) Enzymatic Quality assessments (i) Maintenance of ECM components and architecture: SEM (ii) Acellularity/absence of nuclear content: SEM (iii) Maintenance of vasculature: ultrasound images/acoustic angiography	(i) 130 × 10^6^ human hepatoblast-like cells, Hep3B cells	A nondestructive method, based on the analysis of ultrasound images, is presented in order to evaluate the microvascularization in the decellularized tissue. Recellularized matrix presented liver-specific functions

Mirmalek-Sani et al., USA [21]	Porcine (20-25 kg)	Protocol (i) Chemical Quality assessments (i) Maintenance of ECM components and architecture: SEM and immunostaining (ii) Acellularity/absence of nuclear content: histological evaluation (iii) Maintenance of vasculature: SEM	(i) Hepatoblastoma cells (HepG2) (ii) Total number of cells and infusion rate are not provided by the author	The protocol used for rats was adapted for application in a large animal model, with adequate preservation of essential ECM proteins for cell engraftment and function, as well as the vasculature required for nutrient distribution for whole-organ reseeding. Also showed nonimmunogenicity from decellularized matrices

Yagi et al., Japan [22]	Porcine (20-23 kg)	Protocol (i) Physical (ii) Chemical Quality assessments (i) Maintenance of ECM components and architecture: histological evaluation, immunostaining, and SEM (ii) Acellularity/absence of nuclear content: DAPI and DNA content quantification (iii) Maintenance of vasculature: vascular corrosion casting and digital radiography (iv) Biochemical parameters: immunohistochemical for HGF, bFGF, VEGF, and IGF-1	(i) 1 × 10^9^ porcine hepatocytes were seeded by the multistep infusion method	The authors adapted protocols to be successfully applied in large-scale livers. Engraftment efficiency reported was approximately 74%

Kadota et al., Japan [15]	Lewis rats (200–500 g)	Protocol (i) Physical (ii) Chemical (iii) Enzymatic Quality assessments Not reported	(i) Coinfusion of primary hepatocytes and MSCs (ii) Different cell numbers tested: 3 × 10^8^, 1 × 10^8^ and 5 × 10^7^, with 20% MSCs in different conditions	Authors suggest that MSCs act as supportive cells in this system improving the functionality of the protein production in the engineered tissue

Jiang et al., Taiwan [20]	Balb/c mice (10–30 g)	Protocol (i) Physical (ii) Chemical Quality assessments (i) Maintenance of ECM components and architecture: immunostaining and histological analyses (ii) Acellularity/absence of nuclear content: phase contrast microscopy, DNA content assay, and SEM	(i) 50 × 10^6^ MSC undifferentiated or submitted to prior in vitro stimulation for hepatic differentiation (ii) Transplantation in chemical liver-injured mice	It provides evidence for increased hepatic differentiation of MSC in the decellularized scaffold when compared to 2D culture

Struecker et al., Germany [23]	Porcine (20-60 kg)	Protocol (i) Chemical Quality assessments (i) Maintenance of ECM components and architecture: immunostaining and GAG and collagen content quantification (ii) Acellularity/absence of nuclear content: DNA content quantification (iii) Maintenance of vasculature: computed tomography	Not performed	It presents a fast and effective method by inserting pressure gradients in the perfusion protocol, which improves the homogeneity of perfusion and the outcome of decellularization

Sabetkish et al., Iran [19]	Sprague-Dawley rat (250–300 g) and sheep (not reported)	Protocol (i) Chemical Quality assessments (i) Maintenance of ECM components and architecture: histological evaluation, immunostaining, tensile test, and collagen content (ii) Acellularity/absence of nuclear content: DAPI staining, SEM, and DNA content assay	(i) 18 × 10^6^ GFP primary hepatocytes (ii) Histological evaluation, enzyme tests, and immunofluorescence were performed	By comparing different methods, the authors conclude that using Triton X for the decellularization method is more efficient in maintaining the ultrastructure and biomechanical properties of the tissue. Additionally, it shows that seeding the bioscaffold with cells from the same species is more efficient than xenotransplantation

Wang et al., China [25]	Bama miniature pigs (12-15 kg)	Protocol (i) Physical (ii) Chemical Quality assessments (i) Maintenance of ECM components and architecture: immunostaining, GAC, and collagen content quantification (ii) Acellularity/absence of nuclear content: SEM, DNA content quantification, and H&E staining	Not performed	The study evaluated different methods and defines SDS as the most efficient and fast agent

Mazza et al., England [26]	Human (lobes = 374 g–250 g) (whole liver = 1774 kg)	Protocol (i) Physical (ii) Chemical (iii) Enzymatic Quality assessments (i) Maintenance of ECM components and architecture: collagen and elastin quantification (ii) Acellularity/absence of nuclear content: DNA content quantification, histological evaluation, and DAPI staining	(i) 2 × 10^6^ human hepatic cell lines were used: LX2 (hepatic stellate), HepG2, and Sk-Hep-1 cells (hepatocellular carcinoma)	The author was the first to adapt the protocol of decellularization and recellularization in human liver tissue

Maghsoudlou et al., England [17]	Sprague-Dawley rats (250 g–300 g)	Protocol (i) Chemical (ii) Enzymatic Quality assessments (i) Maintenance of ECM components and architecture: immunostaining (ii) Acellularity/absence of nuclear content: DNA content quantification, H&E staining, and SEM (iii) Maintenance of vasculature: Trypan blue perfusion	(i) 2 × 10^6^ cells (HepG2—human hepatoma cell line)	The protocol of decellularization was optimized with the addition of EDTA to the detergent-enzymatic treatment, creating a denser and more compact matrix

Zhou et al., China [18]	Sprague-Dawley rats (300–350 g)	Protocol (i) Chemical Quality assessments (i) Maintenance of ECM components and architecture: immunostaining and GAG content assay (ii) Acellularity/absence of nuclear content: DNA content assay and SEM	(i) 2 × 10^7^ cells (BRL rat liver cell line)	A method of liver decellularization by continuous perfusion of EDTA and Triton X-100/ammonium hydroxide followed by recellularization with hepatocytes and endothelial cells, showing reendothelization

Coronado et al., USA [24]	Porcine (not reported)	Protocol (i) Chemical Quality assessments (i) Maintenance of ECM components and architecture: protein content quantification (ii) Acellularity/absence of nuclear content: DNA content quantification, histological evaluation, and DAPI staining (iii) Biochemical parameters: HGF/FGF/EGF	(i) Primary porcine hepatocytes (ii) Porcine bioscaffold was lyophilized for cell seeding	Two different decellularization methods were performed in the porcine liver. The method using ammonia and acetic acid was the most efficient in the removal of genetic material. Hepatocytes presented better functionality when seeded in liver substrate (comparing to collagen I)

Mazza et al., England [27]	Human (not reported)	Protocol (i) Physical (ii) Chemical (iii) Enzymatic Quality assessments (i) Maintenance of ECM components and architecture: proteomic, histological, and immunostaining analyses (ii) Acellularity/absence of nuclear content: DNA quantification (iii) Maintenance of vasculature: SEM imaging, confocal autofluorescence microscopy, and chicken egg chorioallantoic membrane (CAM) assay	(i) 1.4-2 × 10^6^ LX2, HepG2, HUVEC, and primary cells (hepatocytes and stellate cells)	Optimization of the previous work, the protocol consists in g-force oscillation and high shear stress to produce acellular liver cubes, using human liver tissue, followed by recellularization

Robertson et al., USA [10]	Sprague-Dawley rats (250–300 g)	Protocol (i) Chemical (ii) Enzymatic Quality assessments (i) Maintenance of ECM components and architecture: immunostaining and GAG content quantification (ii) Acellularity/absence of nuclear content: DNA content by Hoechst 33258 staining and SEM	(i) 1-20 × 10^6^ rat liver cells or 20 × 10^6^ human liver cells	Decellularization method using SDS+DNase was associated with the lowest amount of residual DNA and the highest retention of GAGs. Advances in the recellularization method by reseeding human liver cells in a rat bioscaffold and maintaining the bioengineered tissue for 28 days
